# Omicron variant showed lower neutralizing sensitivity than other SARS-CoV-2 variants to immune sera elicited by vaccines after boost

**DOI:** 10.1080/22221751.2021.2022440

**Published:** 2022-01-24

**Authors:** Jingwen Ai, Haocheng Zhang, Yi Zhang, Ke Lin, Yanliang Zhang, Jing Wu, Yanming Wan, Yanfang Huang, Jieyu Song, Zhangfan Fu, Hongyu Wang, Jingxin Guo, Ning Jiang, Mingxiang Fan, Yang Zhou, Yuanhan Zhao, Qiran Zhang, Qiang Liu, Jing Lv, Peiyao Li, Chao Qiu, Wenhong Zhang

**Affiliations:** a Department of Infectious Disease of Huashan Hospital, National Medical Center for Infectious Diseases and Shanghai Key Laboratory of Infectious Diseases and Biosafety Emergency Response, Fudan University, Shanghai, People’s Republic of China; b Depatment of Infectious Disease, Nanjing Hospital of Chinese Medicine Affiliated to Nanjing University of Chinese Medicine, Jiangsu, People’s Republic of China; c Gobond Testing Technology (Beijing) Co., Ltd, Beijing, People’s Republic of China; d Key Laboratory of Medical Molecular Virology (MOE/MOH) and Institutes of Biomedical Sciences, Shanghai Medical College, Fudan University, Shanghai, People’s Republic of China; e State Key Laboratory of Genetic Engineering, School of Life Science, Fudan University, Shanghai, People’s Republic of China; f National Clinical Research Centre for Aging and Medicine, Huashan Hospital, Fudan University, Shanghai, People’s Republic of China

**Keywords:** Omicron, Covid-19, neutralizing antibody, Pseudotyped viruses, vaccine

## Abstract

The emerging new VOC B.1.1.529 (Omicron) variant has raised serious concerns due to multiple mutations, reported significant immune escape, and unprecedented rapid spreading speed. Currently, studies describing the neutralization ability of different homologous and heterologous booster vaccination against Omicron are still lacking. In this study, we explored the immunogenicity of COVID-19 breakthrough patients, BBIBP-CorV homologous booster group and BBIBP-CorV/ZF2001 heterologous booster group against SARS-CoV-2 pseudotypes corresponding to the prototype, Beta, Delta, and the emergent Omicron variant.

Notably, at 14 days post two-dose inactivated vaccines, pVNT titre increased to 67.4 GMTs against prototype, 8.85 against Beta and 35.07 against Delta, while neutralization activity against Omicron was below the lower limit of quantitation in 80% of the samples. At day 14 post BBIBP-CorV homologous booster vaccination, GMTs of pVNT significantly increased to 285.6, 215.7, 250.8, 48.73 against prototype, Beta, Delta, and Omicron, while at day 14 post ZF2001 heterologous booster vaccination, GMTs of pVNT significantly increased to 1436.00, 789.6, 1501.00, 95.86, respectively. Post booster vaccination, 100% samples showed positive neutralization activity against Omicron, albeit illustrated a significant reduction (5.86- to 14.98-fold) of pVNT against Omicron compared to prototype at 14 days after the homologous or heterologous vaccine boosters.

Overall, our study demonstrates that vaccine-induced immune protection might more likely be escaped by Omicron compared to prototypes and other VOCs. After two doses of inactivated whole-virion vaccines as the “priming” shot, a third heterologous protein subunit vaccine and a homologous inactivated vaccine booster could improve neutralization against Omicron.

## Introduction

The B.1.1.529 (Omicron) variant was first identified in South Africa on November 9^th^, 2021. It was reported to the WHO on November 24^th^ because of the sharp increase of 77 Omicron cases in Gauteng and was later defined as a new VOC-Omicron on November 26^th^. As of December 20^th^, Omicron has been detected in 51 countries and regions globally, and the total number of Omicron sequences has been deposited to the public database yet exceeded 7000. Since November 29^th^, the confirmed COVID-19 cases have increased exponentially in South Africa, showing a trend of rapid spreading and characteristics of high basic reproduction number (R0), and the highest daily new confirmed cases have reached over 37000.

The Omicron variant has quickly raised serious concerns globally, mainly because it has over 50 mutations, of which over 30 mutations were in spike protein. Among these, fifteen mutations are in the receptor-binding domain (RBD), which is responsible for interacting with the ACE2 receptor. Evidence led to the suspicions that Omicron may have evolved to significant immune escape, the higher chance of SARS-CoV-2 reinfection, and unprecedented rapid spreading speed.

Till now, there have been a few pieces of evidence showing that Omicron could lead to a 10–40 -fold reduction in neutralization capacity, and it could evade the binding of most but not all licenced monoclonal antibodies directly interfering with the interaction between ACE2 and spike, like LY-CoV555, LY−CoV016, REGN10933, AZD1061, etc. A recent study showed the secondary breakthrough infection rate of household confirmed Omicron was about 21.6%, which is twice that of the Delta Variant.

However, there is still a lack of a systematic description of the neutralization capacity of breakthrough infection patient plasma, and different homologous and heterologous booster vaccination against Omicron. Here, we explored the immunogenicity of COVID-19 breakthrough infection patients, a third heterologous booster of protein subunit vaccine (ZF2001) primed with two doses of inactivated vaccines, and a third homologous booster of an inactivated vaccine against SARS-CoV-2 pseudotypes corresponding to the prototype, Beta, Delta, and the emergent Omicron variant.

## Methods

**Figure 1.**
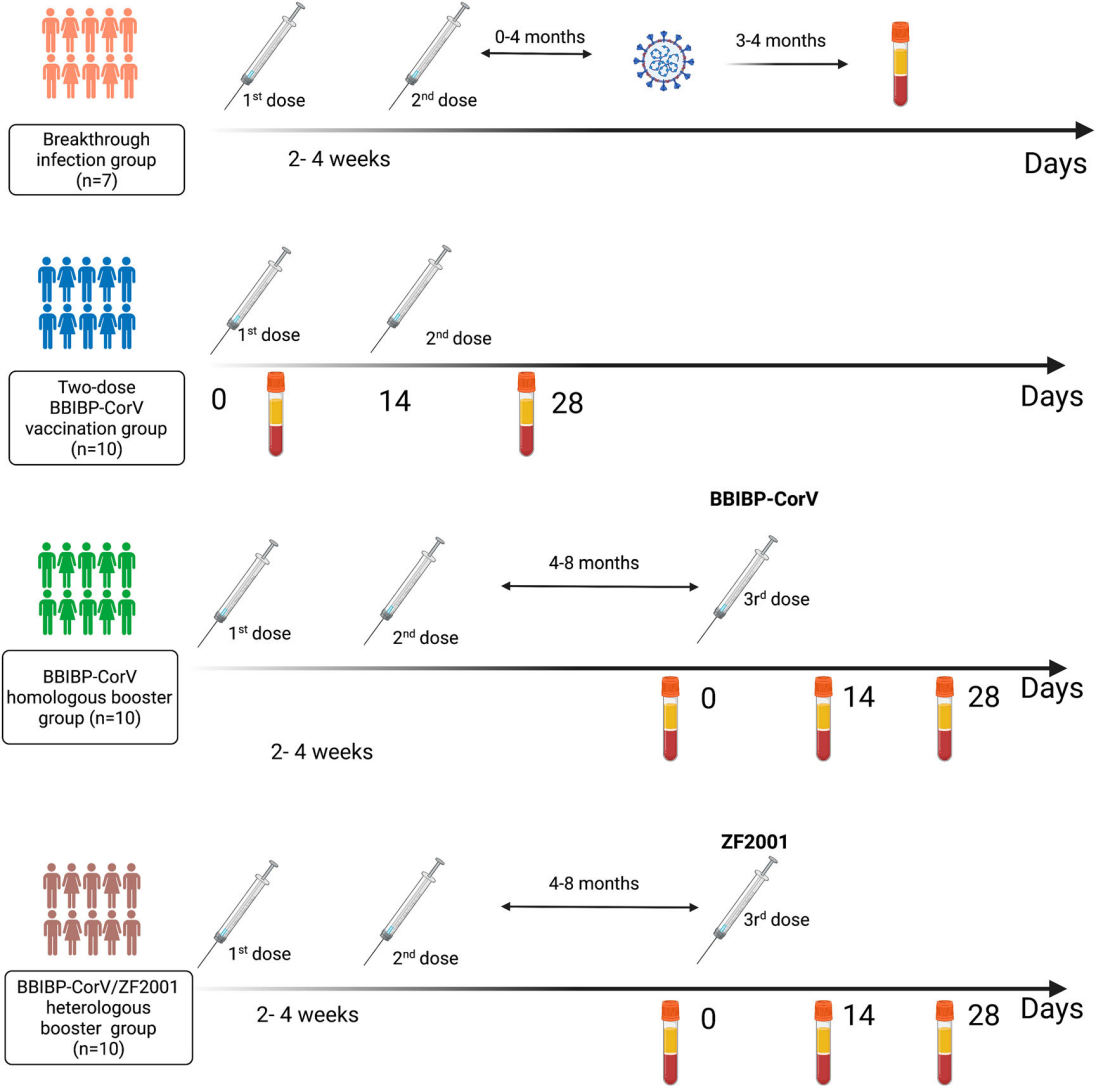
**The details of breakthrough infection and vaccine groups plasma samples collected in our study.** The four panels refer to 1) breakthrough infection group; 2) Two-dose BBIBP-CorV vaccination group; 3) BBIBP-CorV homologous booster group; 4) BBIBP-CorV/ZF2001 heterologous booster group.

We performed neutralizing activity evaluation using convalescent and vaccine plasma in four groups. In the breakthrough infection group, plasma samples were collected from 7 participants after 3–4 months of SARS-CoV-2 breakthrough infection caused by Delta Variant in July 2021. All participants were immunized with two-dose inactivated vaccines (CoronaVac) pre-infection **(**Figure 1**)**. In the two-dose BBIBP-CorV vaccination group, unvaccinated samples and plasmas were collected 14 days post the second vaccination. For BBIBP-CorV homologous booster group (n = 10) and BBIBP-CorV/ ZF2001 heterologous booster group(n = 10), plasma samples were collected at 4–8 months (n = 10) post two-dose BBIBP-CorV vaccination, 14 and 28 days post a third homologous BBIBP-CorV and heterologous ZF2001 booster vaccination **(**Figure 1**)**.

Pseudotyped viruses were produced by 293T cells, both transfected with S protein-expressing plasmid (prototype virus, Beta, Delta, and Omicron variants) and infected with VSV G pseudotyped virus (G*ΔG-VSV) [1]. The plasma pseudovirus neutralization test (pVNT) was carried out to detect neutralization titres. The Pseudotyped viruses used in neutralization titre detection and their mutation site of S genes were listed in the Supplementary Table 1. The initial dilution was 1:10 followed by several dilutions, and the final dilution of the sample was 1: 21870. The limitation of the detection (LOD) was 1:10. Results below the LOD were set to 0.5 times of the LOD. The study was conducted according to the guidelines of the Declaration of Helsinki and approved by the Institutional Review Board of the Ethics Committee of Huashan Hospital (2021-041 and 2021-749) and Nanjing Hospital of Chinese Medicine (2021162). All the participants provided written informed consents.

**Table 1.** Basic characteristics of participants from four cohorts, including age, gender and body mass index. BMI, body mass index. CAD, coronary artery disease. HTN, hypertension. DM, diabetes mellitus. Arrhy, arrhythmia, NASH, non-alcoholic steatohepatitis.NA, not available.

	Breakthrough infection group (n = 7)	Two-dose BBIBP-CorV vaccination group (n = 10)	BBIBP-CorV homologous booster group (n = 10)	BBIBP-CorV/ ZF2001 heterologous booster group (n = 10)	*P* value
**Age (years), median (range)**	45 (34-54)	34 (22-47)	27 (20-51)	24.5 (20-45)	<0.001
**Male, n (%)**	3 (42.9%)	5 (50.0%)	6 (60.0%)	6 (60.0%)	0.912
**BMI (kg/m^2^), mean (SD)**	23.51 (5.296)	22.33 (2.584)	24.16 (4.073)	23.21 (2.828)	0.743
**Comorbidities (%)**					
Any, n (%)	2 (28.6%)	0 (0%)	1 (10%)	0 (0%)	0.132
CAD, n (%)	0 (0%)	0 (0%)	0 (0%)	0 (0%)	–
HTN, n (%)	2 (28.6%)	0 (0%)	0 (0%)	0 (0%)	0.032
DM, n (%)	0 (0%)	0 (0%)	0 (0%)	0 (0%)	–
Arrhy, n (%)	0 (0%)	0 (0%)	1 (10%)	0 (0%)	>0.999
NASH, n (%)	0 (0%)	0 (0%)	0 (0%)	0 (0%)	–
Asthma, n (%)	0 (0%)	0 (0%)	0 (0%)	0 (0%)	–
Rhinitis, n (%)	0 (0%)	0 (0%)	0 (0%)	0 (0%)	–
Goiter, n (%)	0 (0%)	0 (0%)	0 (0%)	0 (0%)	–
Urticaria, n (%)	0 (0%)	0 (0%)	0 (0%)	0 (0%)	–

We present summary statistics for individuals’ geometric mean with 95% confidence intervals (CIs). Mann–Whitney U test, Student’s t-test, and One-way ANOVA test was used for continuous variables and Pearson χ² test or Fisher’s exact test for categorical variables to assess the statistical significance between groups and subgroups. Hypothesis testing was two-sided and *P* values of less than 0.05 were significant. IBM SPSS (version 20.0) and Graphpad Prism (version 9.2) were used for statistical analysis.

## Results

Thirty-seven participants were enrolled in the study. The median age was 45 (range, 34-54) in the breakthrough infection group, 34 (range, 22-47) in the two-dose BBIBP-CorV vaccination group, 27 (range, 20-51) in the BBIBP-CorV homologous booster group, and 24.5 (range, 20-45) in the BBIBP-CorV/ZF2001 heterologous booster group (one-way ANOVA, *P* < 0.001), respectively. Three (42.9%) participants in the breakthrough infection group, five (50.0%) participants in the two-dose BBIBP-CorV vaccination group, six (60%) participants in both the BBIBP-CorV homologous booster group and BBIBP-CorV/ZF2001 heterologous booster group were male (*P *= 0.912). Body mass index (BMI) was similar among the four groups (23.41 vs. 22.33 vs. 24.16 vs. 23.21; *P *= 0.742). Two participants in the breakthrough infection group reported a history of hypertension, and one participant in the BBIBP-CorV homologous booster group reported a history of arrhythmia.

We first studied the neutralization activity of breakthrough infection patient plasma, which showed reduced Omicron neutralization. The geometric mean neutralizing titres (GMTs) against prototype, Beta, Delta, and Omicron variants were 1794.40 (95%CI 982.70-3276.00), 327.80 (117.10-917.70), 1223.05 (600.50-2491.00), and 154.00 (67.29-352.40) respectively **(**Figure 1**)**. Thus, neutralization assays against Omicron represented an 11.65 and 7.94-fold reduction compared to prototype and Delta.

**Figure 2.**
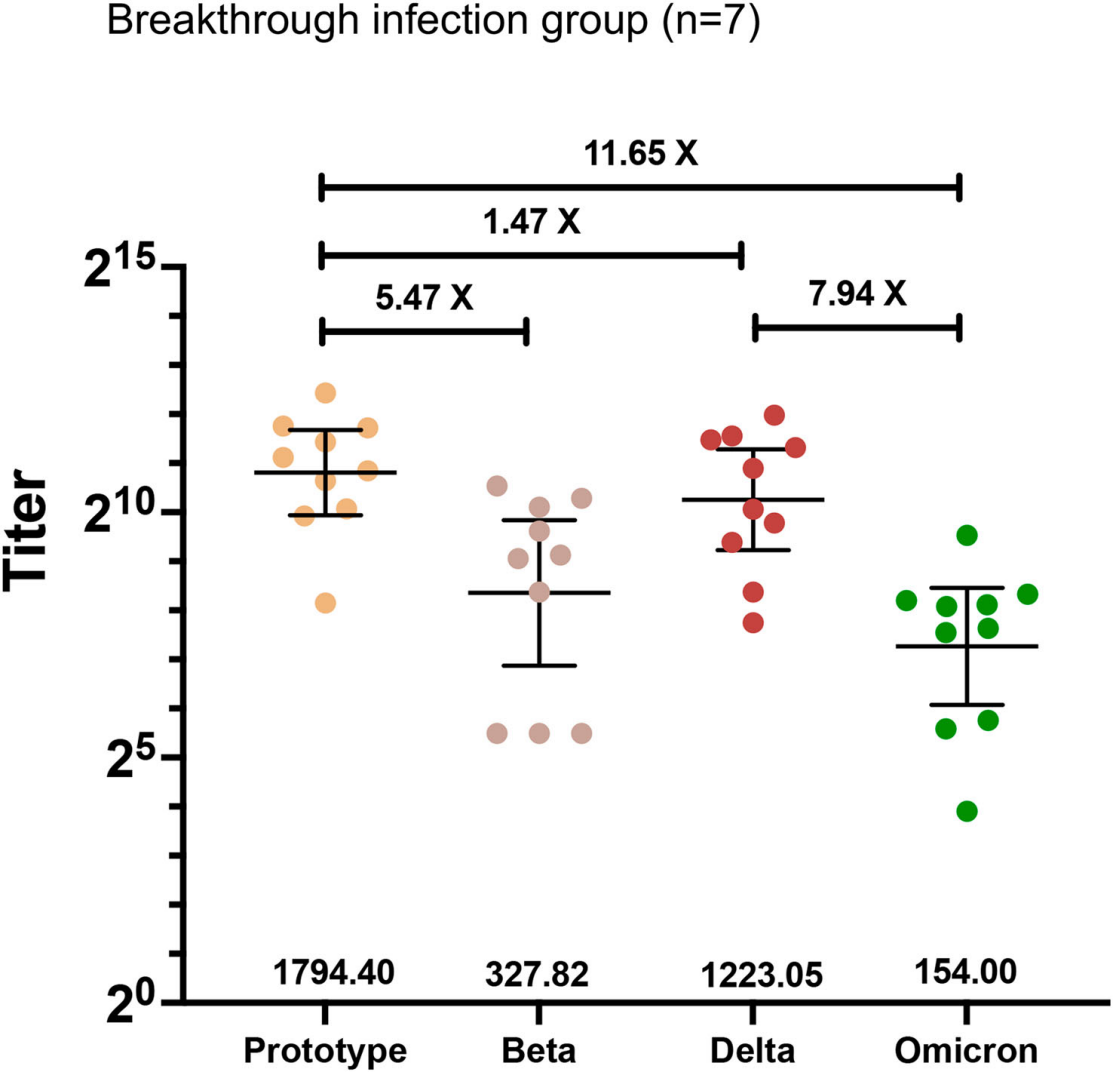
Plasma neutralization titres against Prototype, Beta, Delta, and Omicron SARS-CoV-2 variants in breakthrough infection patients who had previously received two doses inactivated vaccines.

**Figure 3.**
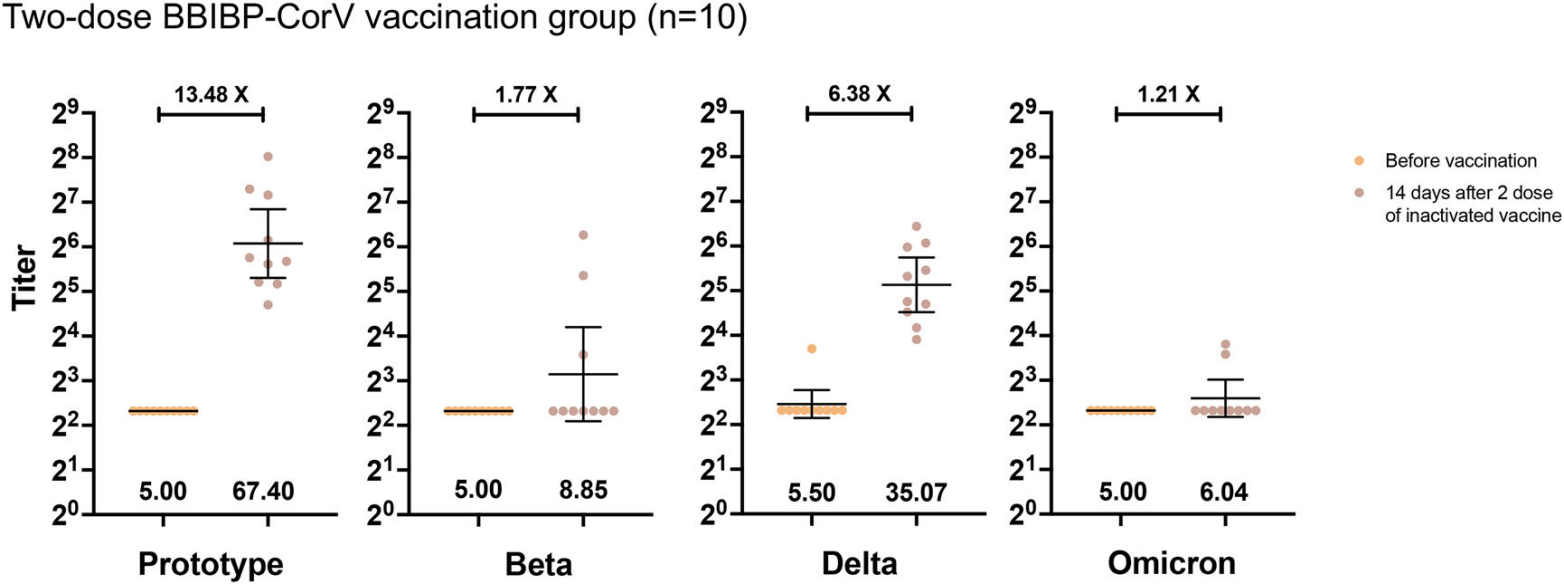
Plasma neutralization titres against Prototype, Beta, Delta, and Omicron SARS-CoV-2 variants in individuals who had received 2-dose inactivated vaccination (neutralization titres evaluated before and 14 days after the second dose).

In the two-dose BBIBP-CorV vaccination group, neutralization activity against Omicron was below the lower limit of quantitation in 80% of the samples at 14 days following the second-dose vaccination of inactivated vaccines. The GMTs were 67.40 (95% CI, 39.56-114.90) against prototype, 8.85 (4.26-18.40) against Beta, 35.07(22.96-53.58) against Delta, and 6.04(4.53-8.07) against Omicron **(**Figure 2**)**. Thus, the neutralization titres of Omicron exhibited a significantly folds reduction than Beta and Delta when compared to the prototype. (11.16-fold vs. 7.62 and 1.92-fold reduction) **(**Figure 3**).** After 4–8 months of two-dose inactivated vaccines (n = 20), the neutralization titres of prototype, Beta, Delta, and Omicron variants were 24.90(95%CI:16.71-37.08), 14.64(10.51-20.40), 29.28(24.39-35.15), and 9.63(6.48-14.32), respectively **(**Supplementary Figure 1 and Supplementary Table 2).

**Figure 4.**
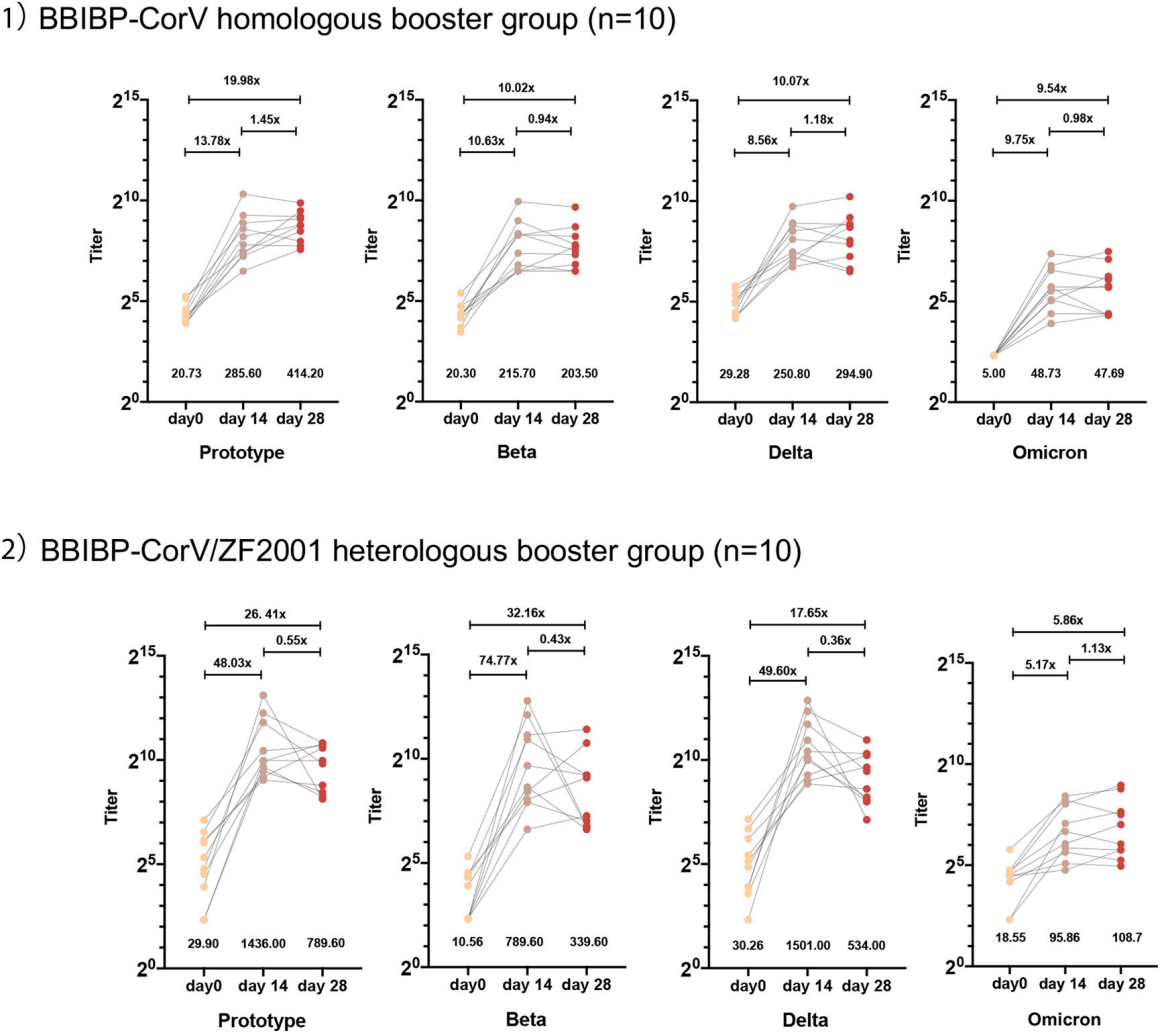
**Plasma neutralization titres against Prototype, Beta, Delta, and Omicron SARS-CoV-2 variants in individuals with booster vaccination dose (neutralization titres evaluated before, 14 and 28 days after the booster dose).** 1) BBIBP-CorV homologous booster group. 2) BBIBP-CorV/ZF2001 heterologous booster group.

For the BBIBP-CorV homologous booster group, at day 14 post booster vaccination with BBIBP-CorV, GMTs of pVNT increased 13.78 (95% CI, 7.71-24.60) folds against prototype (GMT, 20.73 [16.30-26.36] to 285.60 [163.00-500.50]), 10.63(5.62-20.07) folds against Beta (GMT, 20.30[15.47-26.65] to 215.70[118.50-392.60]), 8.56 (4.51-16.26) folds against Delta (GMT, 29.28[21.98-39.02] to 250.80[153.40-410.00]). The neutralization titre against Omicron at day 14 post booster vaccination was 48.73 (28.61-82.99). At day 28 post booster vaccination, GMTs of pVNT stayed stable or increased to 414.20 (284.20-603.60), 203.50(123.90-334.10), 294.90(164.10-529.80), and 47.69(26.37-86.24) against prototype, Beta, Delta and Omicron respectively **(**Figure 4 **a**nd Supplementary Table 2).

A similar trend was observed in the BBIBP-CorV/ZF2001 heterologous booster group. On day 14 post vaccination, GMTs of pVNT increased 48.03 (95% CI, 16.06-143.60) folds against prototype (GMT, 29.90 [13.05-68.49] to 1436.00[716.70-2876.00]), 74.77 (24.85-224.97) folds against Beta (GMT, 10.56[5.856-19.04] to 789.60[288.60-2160.00]), 49.60(14.46-170.05) folds against Delta (GMT, 30.26[14.27-64.18] to 1501.00[752.50-2993.00]), and 5.17(2.52-10.60) folds against Omicron (GMT, 18.55[10.84-31.74] to 95.86[49.82-198.40]). At day 28 post booster vaccination, GMTs of pVNT were 789.60(454.30-1372.00), 339.60(141.20-817.00), 534.00(289.10-986.50), and 108.70(53.56-220.70) against prototype, Beta, Delta and Omicron respectively **(**Figure 4 and Supplementary Table 2).

We illustrated a significant reduction (5.86- to 14.98-fold) of pVNT titre against Omicron compared to the prototype at 14 days after the homologous or heterologous vaccine boosters. The samples of 28 days after booster also exhibited neutralization of Omicron several-fold lower than the prototype. We observed that the GMT of the BBIBP-CorV/ZF2001 heterologous group was relatively higher than that of the BBIBP-CorV homologous group at 14 days (95.86 vs. 48.73) and 28 days (108.70 vs. 47.69) post the boosting vaccination (Supplementary Figure 2). However, the baseline GMT levels are different in these two groups.

## Discussion

In this study, we evaluated the neutralization of the Omicron variant using breakthrough infection patient and vaccinee plasma to answer whether the Omicron variant will evade the immunity barrier previously established by two or three-dose vaccine-elicited immunity or infection. The necessity and urgency to receive a booster vaccination against the new Omicron variant remain unclear.

A few articles show that Omicron has dramatically decreased the potent neutralization ability and reduced the binding affinity of most antibody drugs[2]. The existing study showed Omicron could escape from the majority of current SARS-CoV-2 antibody drugs targeted spike regions, such as LY233 CoV016/LY-CoV555 cocktail, REGN-10933/REGN-109876 cocktail, etc.[3]. The first WHO International standard study showed a 41-fold decrease in the live virus neutralization test against Omicron[4]. The follow-up studies showed the reduction of Omicron was highly variable, from 10 to 25-fold reduction in the pseudotyped neutralization test after two-dose vaccinations[5, 6]. However, the good news is that more and more evidence exhibited that three doses of heterologous or homologous booster vaccination had a 25-100-fold-increase in neutralizing titres compared to two-dose vaccinations[6–8]. And the prior history of infection was associated with high levels of neutralization titres in vaccinated individuals[8].

World Health Organization (WHO) consultation claimed that booster vaccination or additional exposure of vaccinees via infection increases neutralization and may also increase the breadth of neutralizing response[9]. The results were quite comparable to our study. We here reported an elevated neutralization titre against prototype and VOCs in the BBIBP-CorV homologous booster group and the BBIBP-CorV/ZF2001 heterologous booster group compared to the previous two-dose BBIBP-CorV vaccination group, while the breadth of neutralization activity against other variants should be evaluated in the follow-up.

Interestingly, the neutralizing ability after 3-dose booster vaccination against Omicron was comparable to that of the prototype after 2 doses of inactivated vaccines, implying the effectiveness of 3-dose booster vaccination against Omicron may provide similar protection against severe disease and mortality like that of 2-dose vaccinations against prototype.

The reduced neutralization against Omicron compared to prototypes and other VOCs might be associated with mutations in Spike protein. For these single amino acid mutations, E484A and K417N[10–12] both exhibited a high degree of resistance to several monoclonal antibodies. S477N[11] was marked as a lineage-specific mutation in Iota and was been reported immune escape of vaccine-elicited antibody and monoclonal antibodies. Though we here evaluated pVNT of neutralization titres thoroughly, the mutations outside Spike protein should be taken into consideration as well. For a more accurate effect of Omicron on immunity, the vaccine efficacy against the Omicron variant should be evaluated in a real-world study.

Limitations of the study included inaccessibility to live virus neutralization tests. However, we adopted the classic pseudovirus neutralization test commonly used in most published COVID-19 vaccination studies evaluating the immunogenicity of vaccination[13]. The size sample of our studies is still limited, so further larger-scale studies need to be carried out to evaluate immunogenicity and effectiveness of different kinds of a vaccine against Omicron. Single amino acid mutations were not evaluated in our study.

## Conclusion

Overall, our study demonstrates that Omicron might more likely escape vaccine-induced immune protection compared to prototypes and other VOCs. After two doses of inactivated whole-virion vaccines as the “priming” shot, a third heterologous protein subunit vaccine and a homologous inactivated vaccine booster could improve neutralization against Omicron.

## Supplementary Material

Supplemental Material
