# Supplementary material for: Omicron variant showed lower neutralizing sensitivity than other SARS-CoV-2 variants to immune sera elicited by vaccines after boost
Source: Emerg Microbes Infect. 2022 Jan 24;11(1):337–43. doi: 10.1080/22221751.2021.2022440 (PMC8788341; doi:10.1080/22221751.2021.2022440)
Supplement: Supplemental Material [file TEMI_A_2022440_SM0105.docx]

**Supplementary materials**

**Supplementary Table 1.** Pseudotyped viruses used in neutralization titer detection and their mutation site of S genes. VOC, Variants of Concern.

**Supplementary Table 2.** Statistical difference of reduced fold for omicron in four cohorts.

**Supplementary Figure 1.** The pVNT titer of plasma after 4-8 months of two-dose inactivated vaccines

**Supplementary Figure 2.** The pVNT titer against Omicron variant of BBIBP-CorV/ZF2001 heterologous group and BBIBP-CorV homologous group at 14 days and 28 days post the boosting vaccination.

**Supplementary Table 1.** Pseudotyped viruses used in neutralization titer detection and their mutation site of S genes. VOC, Variants of Concern.

| **WHO label** | **Risk Classification** | **Pangolin lineage** | **Pseudotyped virus** | **Mutation site of S gene** (based on sequence Genbank MN908947) |
| --- | --- | --- | --- | --- |
| / | / | / | Prototype | / |
| Beta | VOC | B.1.351 | Beta variant | *L18F, D80A, D215G, delta242-244LAL, R246I, K417N, E484K, N501Y, D614G, A701V* |
| Delta | VOC | B.1.617.2 | Delta variant | *T19R, G142D, E156del, F157del, R158G, L452R, T478K, D614G, P681R, D950N* |
| Omicron | VOC | B.1.1.529 | Omicron variant | *A67V, H69del, V70del, T95I, G142D, V143del, Y144del, Y145del, N211del, L212I, ins214EPE, G339D, S371L, S373P, S375F, K417N, N440K, G446S, S477N, T478K, E484A, Q493R, G496S, Q498R, N501Y, Y505H, T547K, D614G, H655Y, N679K, P681H, N764K, D796Y, N856K, Q954H, N969K, L981F* |

**Supplementary Table 2.** Statistical difference of reduced folds for omicron in four groups.

Breakthrough Infection group (n=7)

| *P* | Prototype | Beta | Delta | Omicron |
| --- | --- | --- | --- | --- |
| Beta | 0.008 | / | / | / |
| Delta | 0.485 | 0.051 | / | / |
| Omicron | <0.001 | 0.203 | 0.001 | / |

Two-dose BBIBP-CorV Vaccination group (Before vaccination vs 14 days after 2 doses of inactivated vaccination) (n=10)

|  | Prototype | Beta | Delta | Omicron |
| --- | --- | --- | --- | --- |
| *P* | <0.001 | 0.211 | <0.001 | 0.474 |

BBIBP-CorV homologous booster group (4-8 months after two-dose vaccination[day0], day14 and day28) (n=10)

| *P* | Prototype | Beta | Delta | Omicron |
| --- | --- | --- | --- | --- |
| Day 14 vs day 0 | <0.001 | <0.001 | <0.001 | <0.001 |
| Day 28 vs day 0 | <0.001 | <0.001 | <0.001 | <0.001 |
| Day 28 vs day 14 | 0.218 | 0.911 | 0.739 | 0.987 |

BBIBP-CorV/ZF2001 heterologous booster group (4-8 months after two-dose vaccination[day0], day14 and day28) (n=10)

| *P* | Prototype | Beta | Delta | Omicron |
| --- | --- | --- | --- | --- |
| Day 14 vs day 0 | <0.001 | <0.001 | <0.001 | <0.001 |
| Day 28 vs day 0 | <0.001 | <0.001 | <0.001 | <0.001 |
| Day 28 vs day 14 | 0.353 | 0.224 | 0.043 | 0.838 |

**Supplementary Figure 1.** The pVNT titer of plasma after 4-8 months of two-dose inactivated vaccines

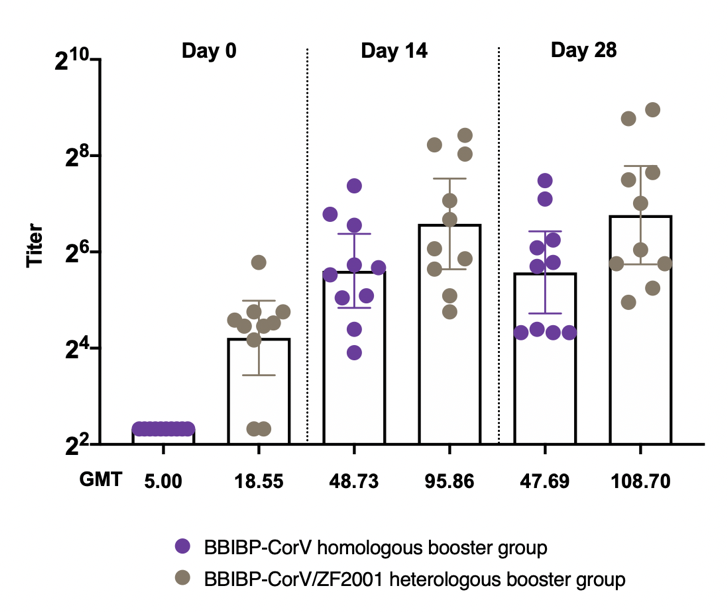
**Supplementary Figure 2.** The pVNT titer against Omicron variant of BBIBP-CorV/ZF2001 heterologous group and BBIBP-CorV homologous group at 14 days and 28 days post the boosting vaccination.
